# Evaluating Acupuncture Point and Nonacupuncture Point Stimulation with EEG: A High-Frequency Power Spectrum Analysis

**DOI:** 10.1155/2016/2134364

**Published:** 2016-10-13

**Authors:** Kwang-Ho Choi, O. Sang Kwon, Seong Jin Cho, Sanghun Lee, Suk-Yun Kang, Seong Hun Ahn, Yeonhee Ryu

**Affiliations:** ^1^KM Fundamental Research Division, Korea Institute of Oriental Medicine, 1672 Yuseong-daero, Yuseong-gu, Daejeon 305-811, Republic of Korea; ^2^Department of Meridian & Acupuncture Point, College of Oriental Medicine, Wonkwang University, 460 Iksan-daero, Iksan, Jeollabuk-do 54538, Republic of Korea

## Abstract

To identify physical and sensory responses to acupuncture point stimulation (APS), nonacupuncture point stimulation (NAPS) and no stimulation (NS), changes in the high-frequency power spectrum before and after stimulation were evaluated with electroencephalography (EEG). A total of 37 healthy subjects received APS at the LI4 point, NAPS, or NS with their eyes closed. Background brain waves were measured before, during, and after stimulation using 8 channels. Changes in the power spectra of gamma waves and high beta waves before, during, and after stimulation were comparatively analyzed. After NAPS, absolute high beta power (AHBP), relative high beta power (RHBP), absolute gamma power (AGP), and relative gamma power (RGP) tended to increase in all channels. But no consistent notable changes were found for APS and NS. NAPS is believed to cause temporary reactions to stress, tension, and sensory responses of the human body, while APS responds stably compared to stimulation of other parts of the body.

## 1. Introduction

Acupuncture points are one of the most fundamental factors in the acupuncture theory of oriental medicine, along with meridian pulses. Efforts have long been made to understand the scientific mechanisms behind acupuncture point and meridian pulse functions. For example, the characteristics of acupuncture points and meridian pulses, including electrical [1], temperature [2], anatomical [3], and photon [4] characteristics, have been identified.

In addition to the direct characteristics of acupuncture points, several studies using bioimaging technology, such as electroencephalography (EEG) and functional magnetic resonance imaging, have evaluated the effects of the physical stimulation of acupuncture points on the human body. Among these technologies, EEG is widely used in clinical evaluation and diagnosis because it is cost effective and provides a simple method for measurement and analysis compared to other tools. Through diverse analyses of EEG data, we can indirectly analogize the neurological functions of human bodies or human responses.

Studies on acupuncture point stimulation using EEG have been reported and include research on alpha and theta wave changes through acupuncture stimulation at the TE5 point [5], the sleep opioid mechanism induced by an electrical needle [6], the activation of specific parts of the cerebral cortex by magnetic stimulation at the PC6 point [7], and a reduction of spectral entropy by finger pressure on acupuncture points [8].

Studies on acupuncture point-related traditional medicine have focused mainly on the features of acupuncture points or human body-related phenomena that occur when acupuncture points are stimulated. Most studies assessing the human response to the stimulation of acupuncture points or the relevant therapeutic effects use sham stimulation, which is considered a placebo, as a control group. There have been few studies to date on the human response when nonacupuncture points are stimulated, although this is not less important. A recent study reported no difference in the impedance between acupuncture point and nonacupuncture point stimulation in healthy subjects [9], but this study was only evaluated to the physical differences between acupuncture points and nonacupuncture points and did not consider the human response to physical stimulation of acupuncture and nonacupuncture points.

Our study ascertained changes in the high-frequency power spectrum through EEG measurement and analysis by carrying out acupuncture point stimulation (APS), nonacupuncture point stimulation (NAPS), and no stimulation (NS) on each subject to evaluate the impact on the human body and the sensory response before and after acupuncture stimulation with each type of stimulation.

## 2. Materials and Methods

### 2.1. Subjects

This study comprised two experiments between July 2010 and August 2011 and targeted healthy male and female subjects in their 20s. The first experiment included 11 males and 7 females, and the second experiment included 10 males and 9 females (Table 1). To ensure a healthy study population, the following exclusion criteria were applied: autoimmune diseases; skin allergies; conditions that can affect the cranial, cervical, and facial electromyogram; surgery or critical medical history within the year prior to the study; metallic implants in the body; medication use before or during the study period; the inability to maintain a sitting position for an hour; contraindications for electrical stimulation; the inability to complete a form; brain-related diseases; and any other factors that the investigator determined to be inappropriate for clinical study.

### 2.2. EEG Measurements

The laboratory was maintained at 24°C and 40% relative humidity using a constant temperature and humidity chamber. Brain waves were measured with a computerized polygraph (PolyG-I, LAXTHA, Korea) with a 256 Hz sampling frequency, 0.7~47 Hz pass filter, and 16-bit analog-to-digital conversion for computer storage. The electrodes were arranged using 8 channels defined by the modified combinatorial nomenclature system. Measurement electrodes were attached at F3, F4, C5, C1, C2, C6, P3, and P4. The reference electrode was attached at the back of the right ear, and the ground electrode was attached at the back of the neck. The electrodes were plate-like discs coated with gold. They were attached using electrode paste and covered with gauze to prevent the paste from drying. For brain wave measurements, the background brain waves were measured 5 times for 3 minutes per measurement while the subjects closed their eyes. It was measured with their eyes closed to prevent possible interference from eye movements. In the 1st experiment, the subjects did not perform any tasks, but in the 2nd experiment subjects were asked to count to themselves while the background brain waves were being measured.

### 2.3. Acupuncture Stimulation

Acupuncture stimulation was performed using APS, NAPS, and NS. All three stimulations were performed for each subject at a minimum of 1-day intervals. The subjects were not told whether the stimulation was APS or NAPS but did know if it was NS. APS was performed at the hapgok (LI4) point of both hands starting with the right hand and using a coated needle that was 40 mm long and 0.25 mm thick (acupuncture needle, Dongbang Acupuncture, Korea). NAPS was performed 1 cm from the LI4 regardless of direction. NS was performed following the same procedure but without stimulation. The presence of pain and the depth of the needle insertion were recorded.

### 2.4. Single-Blind

The subjects were not instructed about acupuncture point stimulation (APS) and nonacupuncture point stimulation (NAPS) before acupuncture stimulation. Also, acupuncture stimulation was performed while the subjects had their eyes closed so that they could not distinguish between APS and NAPS. However, the nonstimulation (NS) experiment did not involve any stimulation so the subjects were not blinded.

### 2.5. Procedures

**Figure 1:**
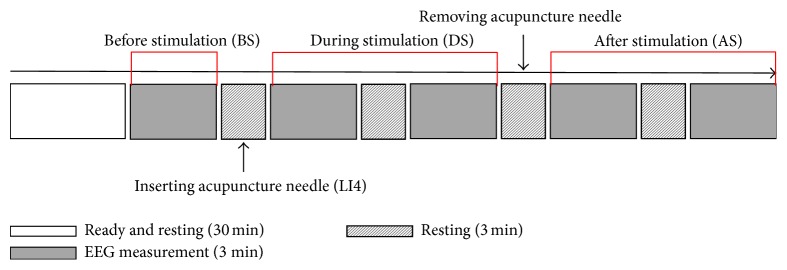
Experimental procedures. After a 30-minute rest, the EEG was performed 5 times for 3 minutes per measurement, with a 3-minute rest time between measurements. During the resting time after the first EEG measurement, an acupuncture needle was inserted at the acupuncture point and removed during the resting time after the third EEG measurement. The analysis was performed by dividing the EEG into 3 parts: before stimulation (BS), during stimulation (DS), and after stimulation (AS).

The experimental procedures for the 1st and 2nd experiments were the same. Prior to the experiment, the subjects rested for 30 minutes. EEG measurements were prepared during the 30-minute rest and began immediately after the rest period. EEG measurements were taken 5 times at 3 minutes per measurement. Between EEG measurements, patients rested for 3 minutes. After the first EEG measurement was completed, an acupuncture needle was inserted during the resting time in the arranged order and, after the third EEG measurement, that needle was removed (Figure 1).

### 2.6. Analysis

The measured data were analyzed using the analysis program (Telescan, LAXTHA, Korea). EEG data were divided into measurements before stimulation (BS), during stimulation (DS), and after stimulation (AS). For each subject, data were selected from the BS, DS, and AS groups, and a total of 30 data points at 2-second intervals were randomly selected from the noiseless regions for fast Fourier transform. The data were then averaged for the power spectrum analysis. To determine the changes in the fast frequency areas, the absolute power and relative power values of high beta power (20~30 Hz) and gamma power (30~47 Hz) were compared by stimulation type. The relative power calculated the proportion of high beta power or gamma power in the entire EEG frequency (0–50 Hz). For statistical analysis, ANOVA was performed to analyze the differences before and after each stimulation.

## 3. Results

### 3.1. First Experiment

**Figure 2:**
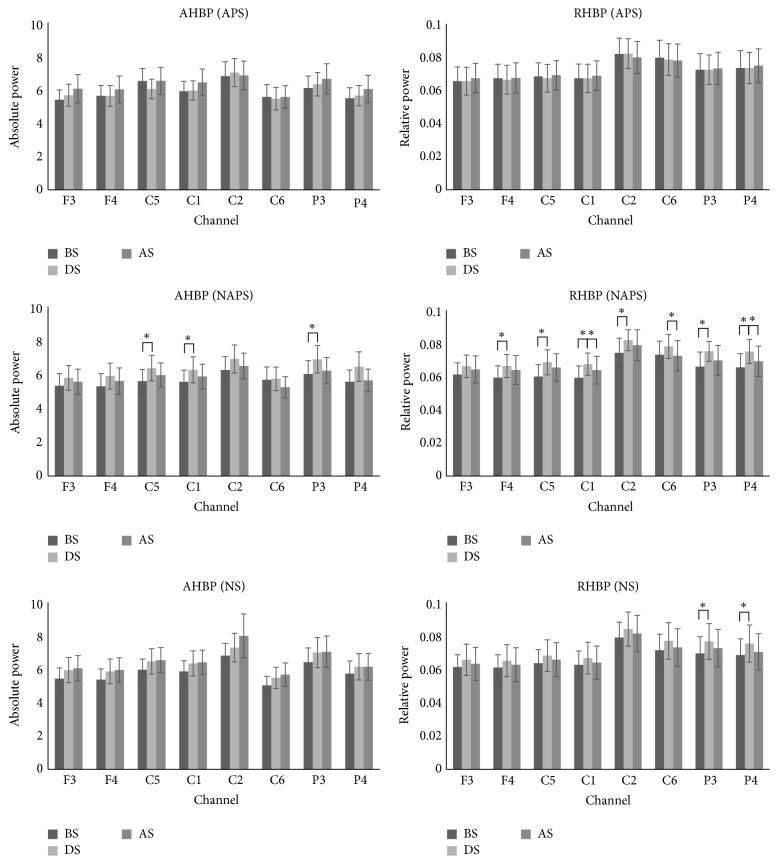
The absolute and relative power of the high beta waves in the 1st experiment (APS, NAPS, and NS). For APS, no distinctive changes were observed. For NAPS, AHBP and RHBP tended to increase in all channels during stimulation and decrease after stimulation, with significant changes in AHBP in 3 channels and in RHBP in 7 channels. For NS, AHBP tended to increase after stimulation compared to before stimulation, and RHBP tended to increase during stimulation and decrease after stimulation, with significant changes in two channels. ^*∗*^ *p* < 0.05.

For APS, no distinctive changes were observed in the high beta waves. For NAPS, AHBP and RHBP increased in all channels during stimulation and tended to decrease after stimulation, with significant changes in AHBP in the C5, C1, and P3 channels and in RHBP in the F4, C5, C1, C2, C6, P3, and P4 channels. For NS, AHBP tended to increase after stimulation compared to before stimulation, whereas RHBP tended to increase during stimulation and then decrease after stimulation, with significant changes in the P3 and P4 channels (Figure 2).

**Figure 3:**
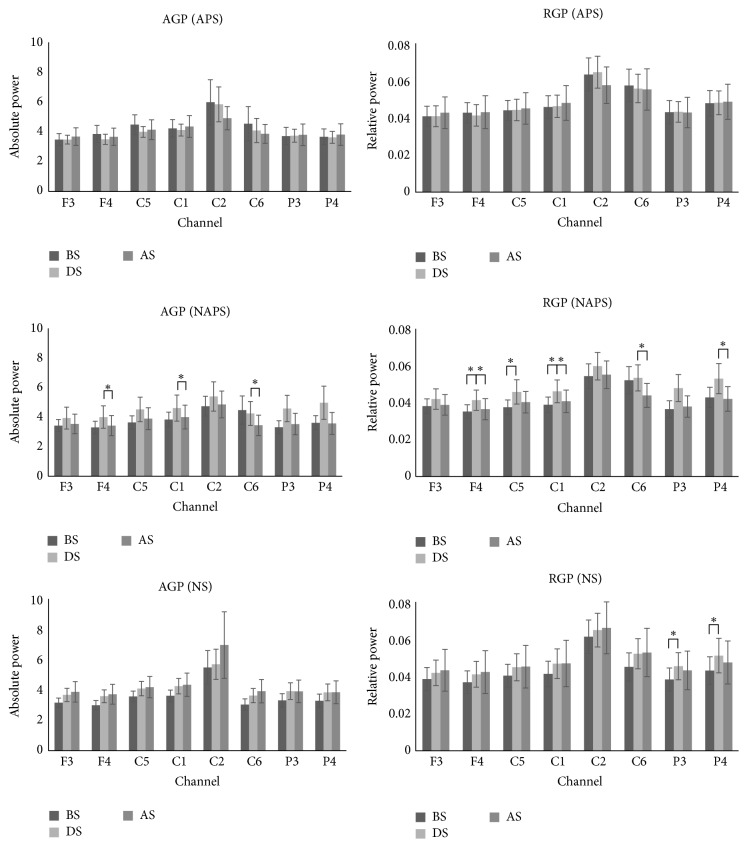
The absolute and relative power of gamma waves in the 1st experiment (APS, NAPS, and NS). For APS, AGP and RGP tended to decrease in only two channels after stimulation, with no changes in the other channels. For NAPS, as in the 1st experiment, AGP and RGP increased in all channels during stimulation but decreased after stimulation, with significant changes in AGP in 3 channels and in RGP in 5 channels. For NS, power significantly increased in two channels during stimulation. ^*∗*^ *p* < 0.05.

In terms of gamma wave change, for APS, AGP and RGP tended to decrease only in the C2 and C6 channels after stimulation and showed no change in the other channels. For NAPS, AGP and RGP increased in all channels during stimulation and decreased after stimulation, with significant changes in AGP in the F4, C1, and C6 channels and in RGP in the F4, C5, C1, C6, and P4 channels. For NS, RGP increased significantly in the P3 and P4 channels during stimulation (Figure 3).

### 3.2. Second Experiment

**Figure 4:**
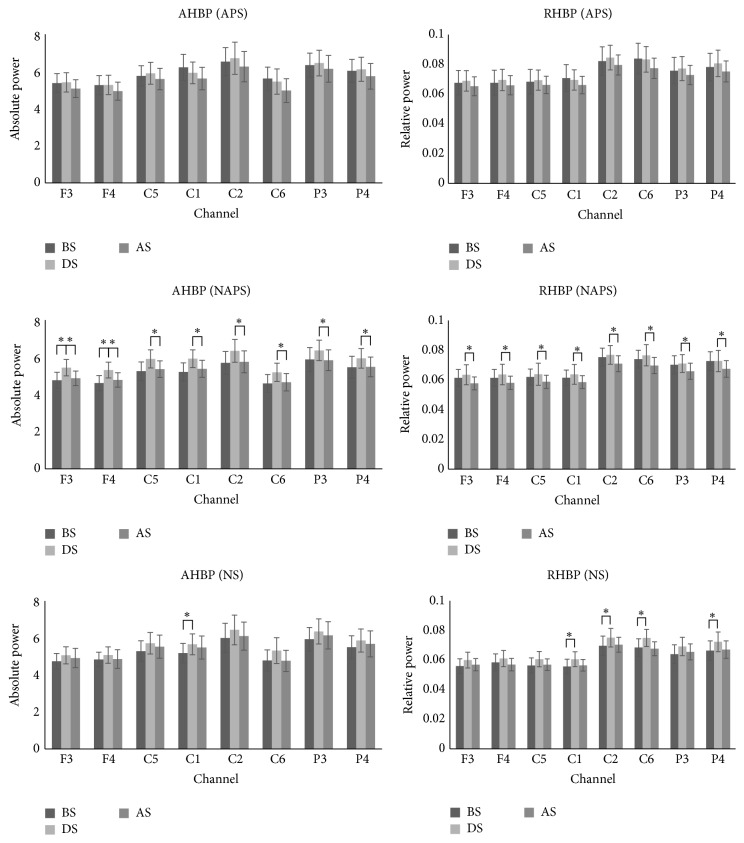
The absolute and relative power of high beta waves in the 2nd experiment (APS, NAPS, and NS). For APS, AHBP and RHBP tended to decrease in all channels after stimulation. For NAPS, as in the 1st experiment, AHBP and RHBP increased in all channels during stimulation but decreased after stimulation, with significant changes in all channels. For NS, values tended to increase during stimulation and decrease after stimulation, with significant changes in absolute power in 1 channel and in relative power in 4 channels. ^*∗*^ *p* < 0.05.

For high beta wave change in APS, AHBP and RHBP tended to decrease in all channels after stimulation. For NAPS, AHBP and RHBP increased in all channels during stimulation and decreased after stimulation. Both AHBP and RHBP showed significant changes in all channels (Figure 4).

**Figure 5:**
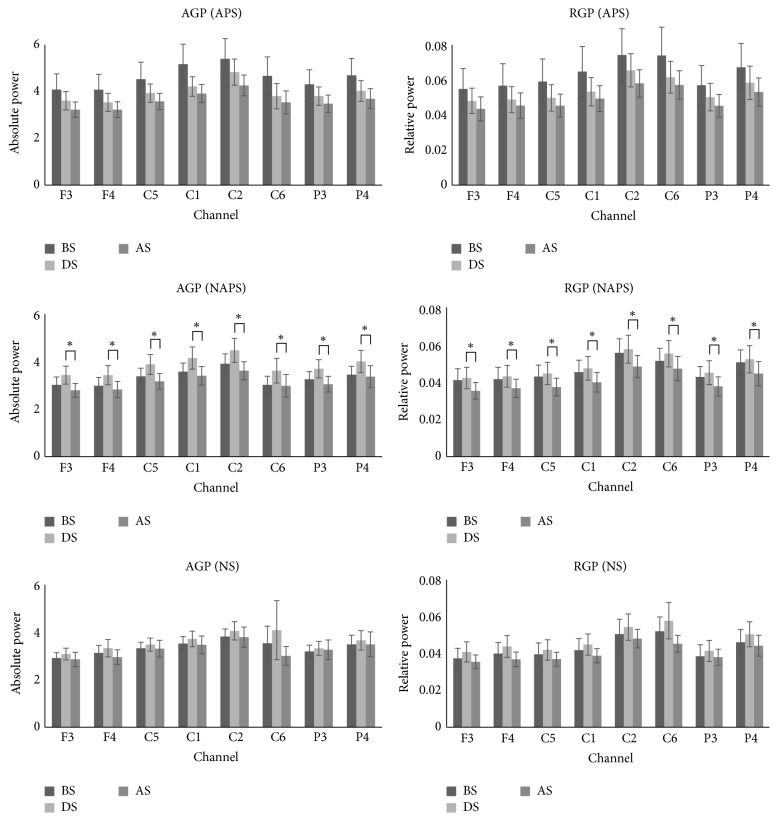
The absolute and relative power of gamma waves in the 2nd experiment (APS, NAPS, and NS). For APS, values tended to decrease in all channels during stimulation and decrease further after stimulation. For NAPS, as in the 1st experiment, AGP and RGP increased in all channels during stimulation but decreased after stimulation, with significant changes in all channels. For NS, a tendency similar to NAPS was observed, but no significant changes were noted. ^*∗*^ *p* < 0.05.

For gamma wave change in APS, AGP and RGP tended to decrease only in the C2 and C6 channels after stimulation and showed no changes in the other channels. For NAPS, AGP and RGP increased in all channels during stimulation and decreased after stimulation, with significant changes in AGP in the F4, C1, and C6 channels and in RGP in the F4, C5, C1, C6, and P4 channels. For NS, RGP significantly increased in the P3 and P4 channels during stimulation (Figure 5).

### 3.3. Combined Experiments

**Figure 6:**
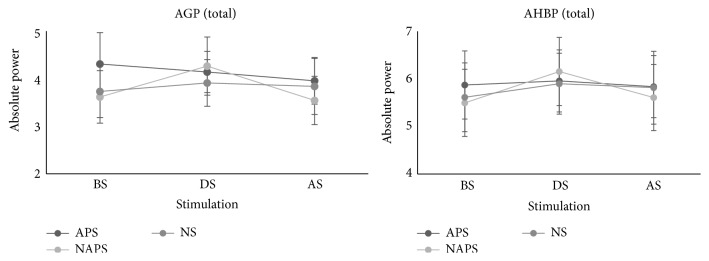
Total channel average of the 1st and 2nd experiments. All data from the 1st and 2nd experiments were averaged for before, during, and after stimulation and increased for NAPS during stimulation.

When the first and second experiments were evaluated together, all parameters increased during stimulation with NAPS but tended to decrease significantly after stimulation, primarily in the parietal lobe region. The average before, during, and after stimulation values in all channels during the first and second experiments showed that AGP and AHBP significantly increased for NAPS during stimulation (Figure 6).

## 4. Discussion

As one of the representative treatments in oriental medicine, acupuncture is widely used around the world and is generally performed by those with an oriental medicine background. The selection of acupuncture points during treatment is very important. In addition to the treatment effect, the direct effect of APS itself on the human body should also be considered. In clinical settings, the effect of acupuncture treatment on patients can be observed easily, but researchers worldwide have tried to investigate the effect of APS to verify this objectively. However, studies on body responses to APS are rare. In addition, the differences between APS and stimulation to parts of the body other than the acupuncture points are not clearly described and have not been studied in detail. Therefore, this study was performed to measure and analyze EEG data before and after APS, NAPS, and NS and to use that data to identify human body responses to acupuncture stimulation.

The experiment was conducted twice, and the most significant changes were experienced in the somatosensory cortex. In the 2nd experiment, the subjects were asked to count to themselves until the end of the experiment both to stay awake and for the consistency of the experiment. In addition, the depth of insertion of the acupuncture needle was measured, and the subjects were asked whether they were experiencing pain.

In the 1st experiment, AHBP, RHBP, AGP, and RGP increased in all channels during stimulation and decreased after stimulation for NAPS. In the 2nd experiment, the results from the 1st experiment were repeated only for NAPS. Significant changes were noted in some channels for NS in the 1st and 2nd experiments, but no common results were obtained from both experiments. Only for NAPS did the two experiments show common results, and channels with significant changes were mainly in the parietal lobe. The averaged results from the 2 experiments and 8 channels showed that the values increased during stimulation only for NAPS (Figure 6).

Other studies reported that EEG measurements with closed eyes can produce deviations [10] and that physical fatigue increases neural activation during the eyes-closed state [11]. It is considered that significant changes in the channels of the occipital area under the NS condition were caused by sleep-inducing the subjects whose brainwaves were repeatedly measured for 30 minutes without stimulation, along with data deviation at each measurement.

High beta wave in EEG has generally been related to stress, and recent studies have shown that addiction [12], anxiety [13], and stress [14, 15] increase high beta waves. Gamma waves, along with beta waves, have been reported to be related to sensory stimulation. One study showed increases in the beta and gamma power by sensory stimulation [16] and another suggested that N30, which is a major index of peripheral sensory cortex reaction in somatosensory evoked potential tests, is related to increases in beta and gamma power [17].

For NAPS, AHBP, RHBP, AGP, and RGP increased in all channels during stimulation in both experiments, suggesting that the greater sensory stimulation of the subjects was induced along with the stress response to acupuncture stimulation. In this study, the depth of needle insertion and the presence of pain after acupuncture stimulation were measured to identify pain responses to APS and NAPS. One interesting finding in the 1st and 2nd experiments is that the acupuncture needle was inserted approximately 0.3~0.6 cm deeper in APS and that the sensation of pain was approximately 16% lower in NAPS in the 1st and 2nd experiments (Tables 2 and 3). Also, the placebo effect was eliminated because the study was conducted in a single-blind to prevent the subjects from distinguishing between APS and NAPS.

This shows that the body responds to acupuncture needles depending on the acupuncture and nonacupuncture points regardless of the degree of actual sensation. Acupuncture stimulation at a location other than acupuncture points can induce tension, stress, and other sensory responses in the body and can yield different results compared to those caused by actual APS. Therefore, during treatment, accurate stimulation should be performed when acupuncture stimulation or other stimulation is applied to acupuncture points to prevent a reduction of treatment effects.

In the previous EEG experiments, changes in the alpha, beta, and gamma bands were observed before and after acupoint stimulation but did not yield consistent results [18], and the presence of acupoints was not acknowledged in anatomy [19]. However, brainwave changes were observed after acupoint stimulation and they occurred in a specific region (occipital lobe) showing similar results as those of visual stimulation [20], thus leaving the results open to controversy.

In this study, the evaluation of body responses to acupuncture stimulation was limited to EEG; therefore, additional research is needed using other body signal measuring tools and other evaluation indices. The study results indirectly show the importance of accurate stimulation on acupuncture points during acupuncture treatment in a clinical situation and suggest that acupuncture treatment at other locations can induce different somatic responses. Based on this, the importance of treatment by APS as performed in oriental medicine is considered to be scientifically verifiable, which will further help improve the quality of treatment.

## 5. Conclusion

Unlike APS, NAPS increases the absolute power and relative power of high beta and gamma waves. Acupuncture stimulation at locations other than acupuncture points is considered to cause temporary responses to stress, tension, or sensory stimulation in the body, while APS stably responds to needle stimulation compared to stimulation to other areas of the body.

## Figures and Tables

**Table 1:** Subject characteristics.

Experiment	Gender (*n*)	Age	Height	Weight
1st	Male (11)	22.76 ± 1.35	174.51 ± 6.19	66.09 ± 6.71
	Female (7)	22.95 ± 2.42	160.14 ± 2.41	51.10 ± 5.74
2nd	Male (10)	24.28 ± 1.11	175.75 ± 7.52	71.59 ± 9.23
	Female (9)	24.42 ± 2.93	160.23 ± 4.52	50.65 ± 5.55

**Table 2:** Depth of acupuncture needle insertion in the 1st and 2nd experiments; APS and NAPS (the depth of needle insertion was greater with APS than with NAPS in both the 1st and 2nd experiments).

Experiment	Stimulation	Depth in right hand (cm)	Depth in left hand (cm)
1st	APS	1.86 ± 0.16	1.88 ± 0.20
	NAPS	1.25 ± 0.13	1.26 ± 0.16
2nd	APS	1.81 ± 0.18	1.71 ± 0.15
	NAPS	1.44 ± 0.32	1.40 ± 0.40

**Table 3:** Number of subjects who felt pain when the acupuncture needle was inserted (the number of subjects who felt pain was higher in the APS group than that of NAPS group).

Experiment	Stimulation	Felt pain (*n*/%)	Did not feel pain (*n*/%)
1st	APS	10/52.63	9/47.37
	NAPS	6/35.29	11/64.71
2nd	APS	11/57.89	8/42.11
	NAPS	8/42.11	11/57.89
